# Reintroduction of confiscated and displaced mammals risks outbreeding and introgression in natural populations, as evidenced by orang-utans of divergent subspecies

**DOI:** 10.1038/srep22026

**Published:** 2016-02-25

**Authors:** Graham L. Banes, Biruté M. F. Galdikas, Linda Vigilant

**Affiliations:** 1 Division of Biological Anthropology, Department of Archaeology and Anthropology, University of Cambridge, Pembroke Street, Cambridge, CB2 3QY, United Kingdom; 2 Max Planck Institute for Evolutionary Anthropology, Deutscher Platz 6, 04103 Leipzig, Germany; 3 CAS-MPG Partner Institute for Computational Biology, 320 Yue Yang Road, Shanghai 200031, People’s Republic of China; 4 Department of Archaeology, Simon Fraser University, 8888 University Drive, Burnaby, B.C., V5A 1S6, Canada

## Abstract

Confiscated and displaced mammals are often taken to sanctuaries, where the explicit goal may be reintroduction to the wild. By inadvertently collecting animals from different source populations, however, such efforts risk reintroducing individuals that have not been in genetic contact for significant periods of time. Using genetic analyses and 44 years of data from Camp Leakey, an orang-utan rehabilitation site on Borneo, we determined the minimum extent to which orang-utans representing non-native, geographically and reproductively isolated taxa were reintroduced into the surrounding wild population. We found two reintroduced females were from a non-native subspecies, and have since produced at least 22 hybridized and introgressed descendants to date, of which at least 15 are living. Given that Bornean orang-utan subspecies are thought to have diverged from a common ancestor around 176,000 years ago, with marked differentiation over the last 80,000 years, we highlight the need for further evaluation of the effects of hybridizing orang-utans of different taxa — particularly in light of the ~1500 displaced orang-utans awaiting urgent reintroduction. As endangered mammals are increasing in number in sanctuaries worldwide, we stress the need for re-examination of historical reintroductions, to assess the extent and effects of *de facto* translocations in the past.

Populations of large and endangered mammals are increasingly impacted by humans, and may live – even in wild or semi-wild settings – under intensive human management (e.g. black-footed ferrets, *Mustela nigripes*^1^; golden lion tamarins, *Leontopithecus rosalia*^2^; northern white rhinos, *Ceratotherium simum cottoni*^3^; giant Galápagos tortoises, *Chelonoidis hoodensis*^4^). Though law enforcement and community engagement have traditionally proven effective for conventional custodial management, more invasive measures are now increasingly needed to ensure the long-term viability of threatened populations^5^,^6^. For example, in Rwanda’s Virunga Massif, close monitoring of individual animals – facilitating the detection and treatment of medical conditions – has led to an annual 4.1%±0.09% increase in the number of mountain gorillas (*Gorilla beringei beringei*) in groups habituated to human observation, versus an annual 0.7%±0.06% decline in the number of unhabituated conspecifics^7^. Proactive captive-breeding of giant pandas, meanwhile, has resulted in the birth of numerous offspring through artificial insemination, producing offspring both for exhibition in zoos and for introduction to the wild – effectively reversing the decline of a conservation flagship species^8^. Though direct intervention may be necessary for the effective conservation of wildlife, unintended effects may ultimately harm populations. For instance, the death of all African wild dogs (*Lycaon pictus*) under study in the Serengeti Ecosystem has been attributed to a rabies vaccination programme, which may have inadvertently compromised dogs’ immune responses through their immobilization, vaccination and radio collaring^9^, though the ill effects of this programme continue to be debated^10^.

Another form of human intervention is placement of endangered animals in sanctuaries. As a result of declining suitable habitat and illegal capture, endangered large mammals are often displaced entirely from wild populations, and may be relocated to sanctuaries for short- or long-term care^11^,^12^. By inadvertently collecting animals from various and potentially unknown points of origin, however, these centres may unintentionally amalgamate individuals from populations that have not been in genetic contact for substantial periods of time. If sanctuaries are in a position to offer lifetime care, with little or no inter-breeding, such mixed genetic origins may not prove to be of importance. Often, however, reintroduction into natural populations is a primary objective of sanctuaries, assuming that concerns regarding disease and suitable habitat are adequately met^12^,^13^,^14^. Given that this can lead to *de facto* translocation, it is important to investigate possible outcomes by looking at deliberate or inadvertent translocations of mammals in the past.

The hybridization of genetically distant individuals may, under some circumstances, increase population fitness^15^,^16^,^17^. For example, the introduction of 8 female panthers from Texas *(Puma concolor stanleyana)* to a remnant population of 50 Florida panthers *(P. c. coryi)* approximately doubled the estimated genetic diversity of the population, and was associated with a marked reduction in the frequency of deleterious traits associated with the breeding of related individuals, such as kinked tails, cryptorchidism and poor sperm quality^18^,^19^,^20^. Measures of fitness and survival improved, and the population tripled in size over 15 years^21^. In grey wolves (*Canis lupus*), the arrival of a single wolf to a bottlenecked Scandinavian population originating from only two individuals was sufficient to dramatically increase genetic diversity (heterozygosity) and exponentially increase population growth^22^. Indeed, the introduction of novel genetic variants into small or genetically depauperate wild populations has been shown, over multiple generations, to augment genetic diversity, reverse indications of inbreeding depression, and increase population size in a range of other animal taxa^23^,^24^,^25^.

Despite this, the hybridization and introgression of representatives of genetically divergent lineages may have negative effects on a population’s overall fitness, as exemplified by the lack of fertility common in offspring resulting from the inter-breeding of different species^26^,^27^,^28^. Reduced fitness, termed outbreeding depression, may also be observed following the hybridization of subspecies, or of other populations that may have independently diverged^26^, particularly if they occupy different habitat types or if no genetic exchange has occurred within the last 500 years^15^. Outbreeding depression can occur via the disruption of gene complexes adapted to local conditions, potentially even giving rise to harmful combinations and/or mutations^28^,^29^. Though there is less empirical evidence for outbreeding depression as compared to inbreeding depression, its consequences are thought to be of a similar magnitude in and after the second generation of offspring^29^. This delay can be attributed to recombination: while first-generation hybrids inherit intact sets of chromosomes from each parental lineage, these chromosomes recombine in subsequent generations, disrupting positive epistatic interactions among parental alleles^28^. Developmental, genetic and other abnormalities have been documented in a wide range of outbred animal taxa, including fish^30^,^31^, invertebrates^32^,^33^, birds^34^ and mammals^35^,^36^ and are further reviewed by Edmands^29^. In some cases, particularly when a population is small, such hybridization and introgression can lead to ‘hybrid swarms’, in which introgressed individuals ‘swamp’ the original population, thus leading to the latter’s extinction^26^. Given that our understanding of outbreeding depression has advanced substantially in recent decades, there is cause for concern that historical reintroductions may have proven deleterious to the fitness of wild populations.

More than 2,650 great apes are confined in sanctuaries across the ranges of all four species: *c.* 963 chimpanzees (*Pan troglodytes*), *c.* 72 bonobos (*P. paniscus*), *c.* 106 gorillas (*Gorilla gorilla*) (Pan African Sanctuary Alliance, pers. comm., 17 January 2016) and *c.* 1516 orang-utans (*Pongo* spp.)^14^. For many sanctuaries, reintroduction is an ultimate goal. The situation is especially pressing for orang-utans, however, given the Indonesian government’s declaration that all sanctuaries should be swiftly emptied through reintroductions, having missed a deadline for doing so by the end of 2015^37^. On the basis of genetic and morphological studies, orang-utans are now known to comprise two species on the islands of Borneo (*Pongo pygmaeus*) and Sumatra (*Pongo abelii*), with three geographically and reproductively isolated subspecies on Borneo: *P. p. wurmbii* in Central and southern West Kalimantan, *P. p. morio* in North and East Kalimantan and Sabah, and *P. p. pygmaeus* in northern West Kalimantan and Sarawak. Each Bornean subspecies is thought to have shared a common ancestor around 176,000 years ago, before radiating to different refugia following climatic fluctuations in the Pleistocene^38^. Particularly marked population differentiation has occurred over the last 80,000 years^39^, with orang-utans currently separated by largely insurmountable river and mountain barriers^38^,^40^,^41^ (Fig. 1). Though not always reciprocally monophyletic^38^,^42^, potentially as the result of human intervention through translocations^42^, subspecies can typically be observed on different clades in a phylogenetic tree of the maternally inherited mitochondrial DNA molecule^43^.

In 1971, however – when Galdikas and Brindamour established their pioneering research and reintroduction site, Camp Leakey, in Tanjung Puting National Park – all orang-utans were classified as a single species, *P. pygmaeus*. Over 14 years, Galdikas and Brindamour rehabilitated and released more than 90 ex-captive or displaced orang-utans into the wild at Camp Leakey, on the southernmost promontory of Central Kalimantan^44^,^45^. Most were confiscated from illegal captivity in the pet trade. Though many were translocated from areas close to the National Park, others came from further afield: though none were thought to have been taken from Sumatra, several were transferred from the provincial capital of Palangka Raya, *c.* 470 kilometres from Tanjung Puting, and others from captivity in Java. In spite of this, Yeager^46^ asserted that at least one juvenile Sumatran female was reintroduced at Camp Leakey prior to 1981^46^, though Galdikas counters that she appeared Bornean in morphology, did not reproduce, and was reported dead by 1994. Further, Galdikas claims to have rejected orang-utans that appeared Sumatran in origin^44^, though she acknowledges that morphology alone is insufficient to diagnose species. Yeager^46^ also suggested that orang-utans from both eastern and western Borneo were probably reintroduced at Camp Leakey, and have potentially inter-bred with the wild population. In spite of this, it is assumed by Galdikas – on the basis of her notes from the 1970s and 1980s regarding individuals’ provenance – that most orang-utans she reintroduced would have been local to Tanjung Puting National Park and the surrounding areas.

Camp Leakey’s contemporary population comprises approximately 60 individuals, including 8 reintroduced female orang-utans. Two further reintroduced females have recently died. All 10 females reproduced; in one case, offspring are now into their fourth generation. As some paternities are known^47^, and as female reproductive success and maternities are well documented as a consequence of four decades of behavioural observation by Galdikas, we determined the taxonomic composition of the orang-utans at Camp Leakey on the basis of mitochondrial DNA. We hypothesised that orang-utans of multiple species or subspecies were translocated and reintroduced, and have since inter-bred with the wild population.

## Methods

Faecal samples were collected from orang-utans in and around the Camp Leakey study area, and genomic DNA extracted, as previously described^47^. Variation in mitochondrial DNA sequences is typically concordant with orang-utan geographic origin, and so sequence analysis of individuals of unknown provenance can aid in inferring their likely origin and taxonomic affiliation^43^. We amplified 397 bp of mitochondrial DNA, primarily spanning a primary stretch of variation in the control region, with the primers Pp-5′ (5′-GCACTTAACTTCACCATC-3′) and Pp-3′ (5′-AAACAAGGGACCACTAAC-3′)^41^. Each 25 μl reaction volume comprised 2.76 μl 10x PCR Buffer (Bioline), 1.03 μl 50 mM MgCl_2_, 1.38 μl 10 mM dNTP mix, 0.83 μl 10 mg/mL BSA, 0.134 μl each primer, 0.28 μl *Taq* polymerase (Life Technologies), 15.7 μl ddH_2_O and 2.75 μl DNA. Thermal cycling conditions were as follows: initial denaturation at 94 °C for 12 minutes; 40 cycles of 94 °C for 40 seconds, 61 °C for 30 seconds and 72 °C for 1 minute; and a final extension at 72 °C for 10 minutes. To avoid amplification of nuclear mitochondrial insertions (‘numts’), which have proved problematic in prior studies of hominoid mtDNA taxa^48^, we also amplified ~1500 bp of mitochondrial DNA in a single PCR – spanning the complete mitochondrial DNA control region – to ensure the resulting longer sequences matched the shorter segments amplified (Banes & Galdikas, in prep.). In all cases, the sequences were concordant and no numts were detected.

Sequences were manually corrected in MEGA 6.06^49^ and aligned using MUSCLE^50^ with published sequences from 20 Bornean and 4 Sumatran orang-utans of known geographic origin (Warren *et al.*, 2001^43^, some updated by Arora *et al.*, 2010^38^). Accession codes for these published sequences are detailed in Supplementary Table S1. Though reportedly generated from amplicons of 278 bp, some published sequences were found to be as short as 164 bp in length, while updated versions were up to 452 bp in length. Consequently, in order to reliably infer a phylogenetic tree, we cut our alignment to 235 bp to encompass the majority of the published sequence data. Phylogenetic trees were inferred using a Bayesian algorithm in MrBAYES 3.2.4^51^ with the HKY + I model of nucleotide substitution, as selected under the Bayesian Information Criterion (BIC) in jModelTest 2.1.6^52^. We applied four independent runs at the default temperature of 0.2, with 10 million generations from a randomly generated tree, sampling every 100 generations. The first 25% of trees generated before convergence were discarded. Sequences that grouped into clades with those of orang-utans of known geographic origin were deemed to have descended ancestrally from those regions, and thus be of the same taxon. This information was combined with parentage data from our prior study^47^ to assess the extent to which reintroduced orang-utans had inter-bred with the wild population.

## Results

Mitochondrial DNA control region sequences were generated from 10 presumed unrelated adult orang-utans (1 male, 9 females) that were reintroduced to the wild at Camp Leakey in the 1970s and 1980s. An additional sequence was obtained from the first-generation offspring (Siswi) of a now-deceased reintroduced female (Siswoyo), and assumed to represent this female given that mtDNA is near-clonally maternally inherited. Of the 11 founders represented, five haplotypes – labelled A to E – were observed in the alignment. Of these, two (A and B) were identical to previously published sequences (GenBank accession codes AJ391121.1 and AJ391108.2, respectively), while three (C, D and E) were novel (GenBank accession codes KU523975-KU523977). Haplotype C featured an insertion that was confirmed by sequencing an additional PCR product amplified from the same DNA extract. Haplotypes D and E each featured a transition, at different bases, when compared with published sequences. These transitions were consistent in all sequences generated from both PCR protocols.

**Figure 1:**
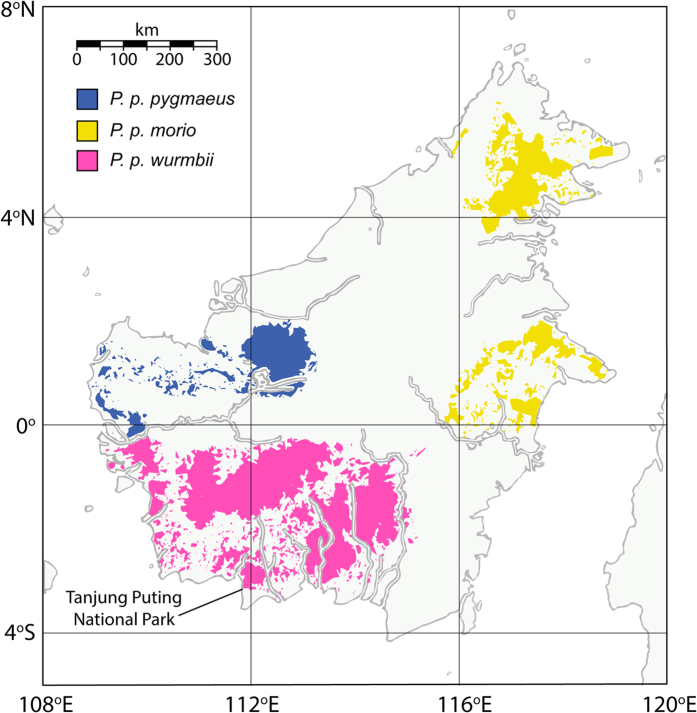
Distribution of orang-utan subspecies on Borneo, in relation to Tanjung Puting National Park. Three subspecies diverged from a common ancestor during climatic fluctuations around 176,000 years ago^38^, with marked population differentiation evolving over the last 80,000 years^39^. *Pongo pygmaeus pygmaeus* is restricted to northern West Kalimantan and Sarawak, and is primarily isolated by the Kapuas River from *P. p. wurmbii*, in southern West and Central Kalimantan. *P. p. morio* is found in East and North Kalimantan and in Sabah, and is isolated by rivers, mountains and geographic distance from the other two subspecies. Figure (including base map) drawn in Adobe Illustrator CC 2015 (http://www.adobe.com/illustrator); orang-utan distribution follows that previously described^77^.

**Figure 2:**
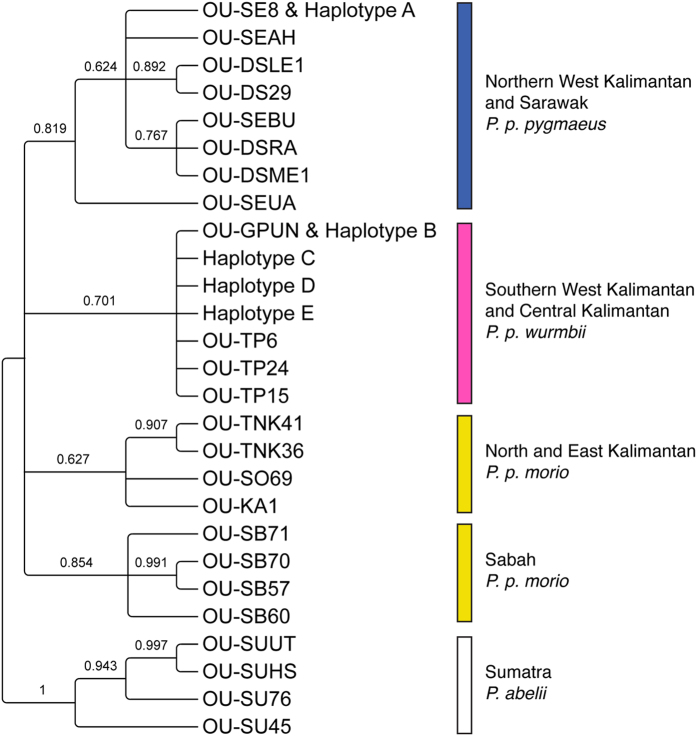
Evolutionary relationships for a 235 bp alignment of 27 unique orang-utan DNA sequences, comprising a primary stretch of variation within the mitochondrial DNA control region that is diagnostic of subspecies. Five haplotypes are represented from Camp Leakey, along with published haplotype sequences from 20 Bornean and 4 Sumatran orang-utans of known geographic origin^38^,^43^.

Phylogenetic analysis placed all five observed haplotypes into clades with Bornean orang-utans of known geographic origin (Fig. 2; Supplementary Table S2). If originally derived from populations in the broad region encompassing Camp Leakey, the five observed haplotypes would be expected to group on the tree with the clade of Bornean orang-utans of *P. p. wurmbii*. We found that nine orang-utan mtDNAs, comprising four haplotypes (B: N = 5, C: N = 1, D: N = 1 and E: N = 2) did indeed fall in this clade. In addition, two females, Rani and Siswoyo, were found to share a haplotype (A) that clustered in the tree with mtDNAs derived from the subspecies *P. p. pygmaeus* from Northern West Kalimantan or Sarawak (Fig. 1).

**Figure 3:**
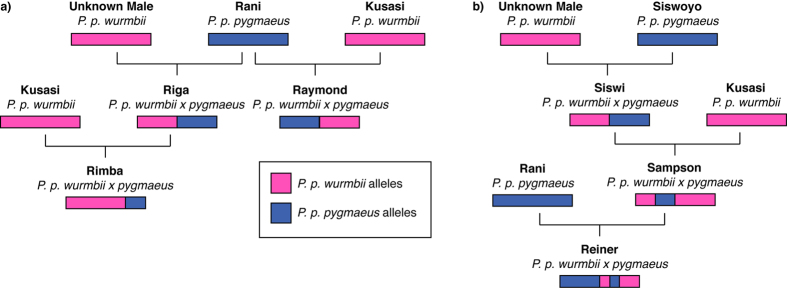
Inter-breeding between Rani (a) and Siswoyo (b) with native males is known to have resulted in hybridized offspring. To illustrate subsequent introgression, this diagram is based on the assumption that 50% of alleles native to each subspecies are always uniformly inherited: thus, by example, ‘Rimba’ is three-quarters *Pongo pygmaeus wurmbii* and one quarter *P. p. pygmaeus.* The figure illustrates examples of the extent of admixture known to have occurred within and between these lineages over multiple generations. In practice, as detailed in the text, Rani’s direct descendants number(ed) at least 14 hybridized and/or introgressed individuals over 3 generations; Siswoyo’s number(ed) at least 8 over 2 generations. At least 9 of their combined descendants are males likely capable of fathering offspring. Their reproductive output is unknown, but has inevitably resulted in further admixture in other lineages.

Both Rani and Siswoyo have bred with native males at Camp Leakey, giving birth to hybridized offspring that – on the basis of paternity and long-term population monitoring – are known to have since introgressed (Fig. 3). Rani (b. ~1969) was reintroduced prior to sexual maturation, having been confiscated in Java. Rani’s descendants include seven first-generation offspring, one of which died, five known second-generation offspring, and two known third-generation offspring, one of which died. With the exception of the two deceased offspring, who both died in infancy, all of Rani’s known descendants are presumed to be alive and none are known to have required veterinary interventions. Siswoyo (b. ~1962, d. 1991) was an adolescent female at the time of her reintroduction, having also been confiscated on Java. Her descendants are comparatively few, with only five first-generation and three second-generation offspring. Two of Siswoyo’s own offspring died in infancy; infection following the latter pregnancy resulted in Siswoyo’s own death ten days post parturition. Her only daughter, Siswi, produced a stillborn offspring, a daughter that died in infancy, and a son that required frequent medical interventions, having been blinded in his right eye after an accident caused by humans in 1993. He was last seen at the age of 16 before disappearing into the forest (Galdikas, unpublished data) and potentially has died. Though in poor condition, Sampson was able to achieve at least one paternity as an unflanged male prior to his departure from the study area^47^. Siswi was given life-saving surgery in 1997 to repair a perforated intestine, and has since been medically unable to conceive (Galdikas, unpublished data).

## Discussion

Our findings provide genetic evidence that individuals of a non-native orang-utan subspecies were translocated and introduced into a wild orang-utan population, and have since hybridized and introgressed over multiple generations. The 22 known descendants of Rani and Siswoyo – of which at least 15 are still alive – can be assumed to carry a ‘cocktail’ of genes that would not normally occur in the wild, comprising maternally inherited mitochondrial DNA specific to *P. p. pygmaeus*, Y-chromosomes inherited from *P. p. wurmbii* fathers, and a mixture of autosomal genes deriving from each subspecies. Such offspring are unlikely to be localised to the Camp Leakey study area: though females are thought to be philopatric, males disperse over vast home ranges that may be larger than 2500 ha^53^. Of Rani’s descendants, at least six are males that could be sexually mature; of Siswoyo’s, at least three might be able to father offspring. It is therefore highly probable that inter-breeding and the production of introgressed offspring is occurring elsewhere in Tanjung Puting National Park, potentially far from Camp Leakey itself, as a consequence of Rani and Siswoyo’s introduction.

Given the size and scale of the reintroduction programme, however, Rani and Siswoyo may simply prove the tip of the iceberg. Galdikas estimates that, of the 90 orang-utans she reintroduced to Camp Leakey, at least 80 were probably alive by the time the programme there ceased, although a number had disappeared into the forest^45^. These include two males and one female that were confiscated from the same owner as Siswoyo, though acquired by him at different times and thus potentially from different sources. These orang-utans might plausibly be *P. p. pygmaeus* or another subspecies entirely. At least 15 reintroduced females at Camp Leakey are known, cumulatively, to have produced at least 78 descendants over up to four generations – of which at least 61 were presumed or known to be living by the end of 2011. With the exception of one adolescent male, all of the deceased offspring were infants, of which at least one was stillborn. It is therefore unclear to what extent the natural population has experienced the introduction of other non-native alleles, which might even incorporate those of Sumatran orang-utans^46^. Yeager’s earlier testimony of Sumatran orang-utans in Tanjung Puting is compounded by the fact that animals are known to have been translocated between the two islands through the pet trade: by genetic testing, two privately owned Bornean individuals were identified in Sumatra in 2006 and 2009, and were consequently repatriated in 2011 for reintroduction into Borneo’s Lamandau Wildlife Reserve (A E Leiman, pers. comm., 1 May 2015). The issue is further confounded by conflicting reports on the extent to which orang-utans were reintroduced into Tanjung Puting. The Indonesian government is known to have released ex-captive orang-utans at two other sites in the National Park throughout the 1980s and 1990s. Galdikas herself reports that, though she ceased reintroduction at Camp Leakey in 1985, she released more than 200 orang-utans into the wider National Park up until 1995, when she ceased all such reintroductions in accordance with changing Indonesian law. Despite this, Yeager^46^ reported that more than 180 orang-utans were released into the wider National Park between 1977 and 1997^46^. Similar claims were made by Spalding, who concurred that many releases took place after 1995, both at Camp Leakey and elsewhere in the National Park^54^,^55^. In the case of all these reintroductions — including those by Galdikas — few records are published or publicly available to specify the exact number of animals released, or when or where those releases occurred. Few attempts have been made to document their outcomes^45^,^56^.

The effects of inter-breeding different orang-utan taxa remain unclear, particularly in reference to their health and reproductive viability. By 1985, such concerns had led zoos to manage their orang-utans as two separate, species-distinct populations^57^, though the potential for outbreeding among Bornean subspecies has yet to receive significant attention. It is noteworthy that Siswoyo and her descendants have experienced poor reproductive success, poor reproductive health, high infant mortality and overall ill health – incidents that might characterise outbreeding depression. By comparison, however, Rani and her descendants have enjoyed one of the greatest reproductive successes of any matriline at the site. Our data are too few, therefore, to determine the extent to which these factors might be linked to their introgression – and, given the slow reproductive rate of orang-utans, which typically reproduce only once every eight years – too few generations of offspring are available to study and potentially correlate these effects. Nonetheless, there is a general consensus in the literature that orang-utan subspecies should not be hybridized in the wild. Arguments against hybridization have ranged from morals and ethics^58^,^59^,^60^ to fears of reduced fertility^61^ and concerns that differing characteristics must be independently preserved^62^,^63^. The genetic effects of outbreeding are of particular concern, however, in light of the substantial differences that have evolved in each subspecies, in morphology, habitat, diet and behavioural ecology^64^ in their tens of thousands of years of independent evolution^39^, all of which may contribute to outbreeding depression^15^.

In spite of this, there is evidence in other primate taxa that hybridization occurs naturally along the borders of different species’ and subspecies’ ranges. Gonder *et al.* (2011) observed a high number of shared private alleles between chimpanzees from the Gulf of Guinea *(Pan troglodytes ellioti)* and from Southern Cameroon *(P. t. verus),* suggesting recent introgression within a purported hybrid zone^65^. Charpentier *et al.* (2012) identified natural hybridization of anubis *(Papio anubis)* and yellow *(P. cynocephalus)* baboons in the Amboseli basin in southern Kenya. This hybridization was notably asymmetric: more anubis baboons dispersed into, and were reproductively successful, within yellow baboon populations than vice-versa^66^. Such asymmetric rates of gene flow have also been observed from rhesus *(Macaca mulatta)* into long-tailed *(M. fascicularis)* macaques^67^ and, ancestrally, from western *(Pan troglodytes verus)* into central *(P. t. troglodytes)* and eastern *(P. t. schweinfurthii)* chimpanzees^68^. Though behavioural explanations for ancestral admixture are speculative, more recent hybridization is thought to suggest an adaptive advantage: baboons with more anubis ancestry might therefore be better adapted for survival among unadmixed yellow baboons, for example^66^. However, in most of the aforementioned cases, the long-term effects of recent hybridization events are unknown, and apparent hybrid vigour may yet give way to outbreeding depression. Furthermore, most hybridization events occurred in the absence of obstructive physical barriers, occurring instead between directly adjacent populations or those separated by an expanse of land that could, in theory, be traversed within an individual’s lifetime. Indeed, some researchers have questioned the validity of the taxonomic units of central (*Pan troglodytes troglodytes*) and eastern (*P. t. schweinfurthii*) chimpanzees, pointing out that the variation in these chimpanzees can be better described as following a pattern of increasing genetic differentiation with increasing distance^69^. In contrast, orang-utan subspecies are separated by largely insurmountable riverine and mountain barriers that limit the potential for large-scale introgression and have ensured independent evolution of the Bornean subspecies for approximately 80,000 years^38^,^39^.

In the decades following the reintroductions at Camp Leakey, extensive guidelines have emerged to better facilitate wildlife reintroductions. Over time, these guidelines have placed more explicit focus on genetic considerations. In their *Guidelines for the Reintroduction of Animals Born or Held in Captivity*, the Association of Zoos and Aquariums suggested that the genotype of reintroduction candidates “should be the closest possible match” to that of the destination population, “so that subspecific distinctions can be maintained”^70^. More recent ‘best practice’ guidelines from the IUCN — including those for the placement of all species of confiscated animals^11^, in addition to those specific to apes^71^,^72^— go substantially further in mandating that genetic testing be performed prior to reintroductions. In the IUCN’s Guidelines for Reintroductions and Other Conservation Translocations, both pre- and post-release genetic monitoring is encouraged, plus the use and banking of non-invasive samples to help measure reintroduction success^12^. Recommendations made to the Indonesian government in 1991^73^ – many of which helped form the basis of the aforementioned guidelines – have also led to significant changes in Indonesian law. In 1995, the Indonesian government mandated that orang-utans can only be reintroduced into areas housing their same subspecies, and that ex-captives cannot be released into forest that is home to extant wild populations (Decree No. 280/KPTS-II/1995).

In practice, however, these guidelines and legislation are challenging to follow or enforce. In many cases, it is simply too expensive to perform genetic testing: rescuing and accommodating displaced animals takes priority in the face of extremely limited funds. The origin of displaced animals might therefore be inferred as the region from which they were rescued (e.g. Tutin *et al.*^74^). A lack of quality laboratory facilities – notably in developing range countries – has also been shown to prove problematic, leading Goossens *et al.*^75^ to call for the development of simpler, cheaper molecular methods that can be employed in basic laboratories by inexperienced personnel^75^. For orang-utans, few rehabilitation centres have performed the required genetic testing in accordance with Indonesian law – those that have done so are thought to have relied solely on mitochondrial DNA, which is uniparentally inherited and thus insufficient to diagnose hybrids – avoiding the costly amplification and use of a geo-referenced panel of autosomal and Y-chromosomal markers (Anonymous 1, pers. comm., 2015). In some cases, orang-utans are known to have been reintroduced into viable wild populations in contravention of the 1995 decree (Anonymous 2, pers. comm., 2015). It is important to acknowledge, however, that efforts to genetically test orang-utans are largely hampered by restrictive Indonesian and Malaysian legislation on the mere collection of biomaterials from endangered species. Further, Indonesia’s domestic legislation on CITES has regulated that faeces be CITES controlled: as a consequence, export of non-invasively collected samples to well-equipped and well-funded laboratories abroad may be time-consuming or impractical. Even with genetic testing, securing protected habitat within the range of each subspecies is proving to be difficult and cost-prohibitive.

Our study demonstrates that reintroduction from sanctuaries can lead – and has led – to *de facto* translocation, which may ultimately have serious consequences for the health and viability of threatened wild populations. As a consequence, we strongly advise adherence to established international guidelines in future reintroductions, given that the effects of such introgression can be applied to a wide range of endangered animal taxa. For orang-utans, however, the issue may require more pressing attention: having failed to meet the terms of an earlier declaration to close all sanctuaries by the end of 2015, policymakers are now exploring their options to urgently meet this objective. Warren’s^76^ suggestion of developing isolated, ‘mixed’ populations of rehabilitant individuals may be an attractive course of action, but would only prove a responsible solution if inter-breeding different orang-utan subspecies can be shown to have no detrimental effects.

## Additional Information

**Accession codes:** Novel sequences can be found in GenBank under the accession codes KU523975-KU523977. 

**How to cite this article**: Banes, G. L. *et al.* Reintroduction of confiscated and displaced mammals risks outbreeding and introgression in natural populations, as evidenced by orang-utans of divergent subspecies. *Sci. Rep.* **6**, 22026; doi: 10.1038/srep22026 (2016).

## Supplementary Material

Supplementary Information
