# Supplementary material for: Reintroduction of confiscated and displaced mammals risks outbreeding and introgression in natural populations, as evidenced by orang-utans of divergent subspecies
Source: Sci Rep. 2016 Feb 25;6:22026. doi: 10.1038/srep22026 (PMC4766574; doi:10.1038/srep22026)
Supplement: Supplementary Information [file srep22026-s1.pdf]

Supplementary Information

Reintroduction of confiscated and displaced mammals risks outbreeding and introgression in natural populations, as evidenced by orang-utans of divergent subspecies

Graham L Banes[123]*, Biruté M F Galdikas[4] and Linda Vigilant[2]

[1] Division of Biological Anthropology, Department of Archaeology and Anthropology, University of Cambridge, Pembroke Street, Cambridge, CB2 3QY, United Kingdom.

[2] Max Planck Institute for Evolutionary Anthropology, Deutscher Platz 6, 04103 Leipzig, Germany.

[3] CAS-MPG Partner Institute for Computational Biology, 320 Yue Yang Road, Shanghai 200031, People's Republic of China.

[4] Department of Archaeology, Simon Fraser University, 8888 University Drive, Burnaby, B.C., V5A 1S6, Canada.

* To whom correspondence should be addressed. E-mail: graham_banes@eva.mpg.de

Table S1 Published sequences from orang-utans of known geographic origin, by Warren *et al.* (2001)[1]. Accession codes correspond to those in GenBank. Sequences with accession codes ending in '2' were updated by Arora *et al.* (2010)[2].

Name	Accession code	Subspecies	Geographic origin
OU-SE8	AJ391121.2	*P. p. pygmaeus*	Semenggoh, Sarawak
OU-SEAH	AJ391122.1	*P. p. pygmaeus*	Semenggoh, Sarawak
OU-DSLE1	AJ391100.2	*P. p. pygmaeus*	Danau Sentarum, Northwest Kalimantan
OU-DS29	AJ391099.1	*P. p. pygmaeus*	Danau Sentarum, Northwest Kalimantan
OU-SEBU	AJ391123.2	*P. p. pygmaeus*	Semenggoh, Sarawak
OU-DSRA	AJ391103.2	*P. p. pygmaeus*	Danau Sentarum, Northwest Kalimantan
OU-DSME1	AJ391101.2	*P. p. pygmaeus*	Danau Sentarum, Northwest Kalimantan
OU-SEUA	AJ391125.2	*P. p. pygmaeus*	Semenggoh, Sarawak
OU-GPUN	AJ391108.2	*P. p. wurmbii*	Gunung Palung, Southwest Kalimantan
OU-TP6	AJ391141.1	*P. p. wurmbii*	Tanjung Puting, Central Kalimantan
OU-TP24	AJ391140.1	*P. p. wurmbii*	Tanjung Puting, Central Kalimantan
OU-TP15	AJ391139.1	*P. p. wurmbii*	Tanjung Puting, Central Kalimantan
OU-TNK41	AJ391137.2	*P. p. morio*	Kutai National Park, East Kalimantan
OU-TNK36	AJ391133.2	*P. p. morio*	Kutai National Park, East Kalimantan
OU-SO69	AJ391126.1	*P. p. morio*	East or North Kalimantan
OU-KA1	AJ391109.2	*P. p. morio*	Sangatta, East Kalimantan
OU-SB71	AJ391120.2	*P. p. morio*	Sandakan, Sabah
OU-SB70	AJ391119.1	*P. p. morio*	Lahad Datu, Sabah
OU-SB57	AJ391117.2	*P. p. morio*	Kinabatangan, Sabah
OU-SB60	AJ391118.2	*P. p. morio*	Kinabatangan, Sabah
OU-SUUT	AJ391131.1	*P. abelii*	Sumatra
OU-SUHS	AJ391130.1	*P. abelii*	Sumatra
OU-SU76	AJ391129.2	*P. abelii*	Bohorok, North Sumatra
OU-SU45	AJ391127.2	*P. abelii*	Bohorok, North Sumatra

Table S2 Positions of nucleotide variation in a 235 bp alignment of the mitochondrial DNA control region, spanning a primary stretch of variation diagnostic of orang-utan subspecies. The alignment comprised 27 unique sequences representing 31 Bornean and 4 Sumatran orang-utans. Only variation among Bornean orang-utans is shown in this table. Highlighted positions denote those potentially useful in inferring orang-utan subspecies.

	**Position in alignment of nucleotide variation**																																							
**DNA sequence**	**7**	**41**	**50**	**56**	**75**	**77**	**78**	**80**	**83**	**86**	**88**	**94**	**95**	**96**	**98**	**99**	**101**	**111**	**117**	**119**	**120**	**121**	**123**	**124**	**133**	**146**	**148**	**153**	**166**	**167**	**168**	**173**	**176**	**184**	**196**	**198**	**201**	**216**	**220**	**228**	**234**
Hap. A	T	T	C	A	C	C	C	C	C	A	T	C	A	T	T	A	T	A	T	C	C	-	C	C	T	C	A	C	C	A	C	C	C	T	T	C	T	A	C	C	C
OU-SEAH	.	.	.	.	.	.	.	.	T	.	.	.	.	.	.	.	.	.	.	.	.	-	.	.	.	.	.	.	.	.	.	.	.	.	.	.	.	.	.	.	.
OU-DSLE1	.	.	.	.	.	.	.	.	.	.	.	.	.	C	.	.	.	.	.	.	.	-	.	.	.	A	.	.	.	.	.	.	.	.	.	.	.	.	.	.	.
OU-DS29	.	.	.	.	.	.	T	.	.	.	.	.	.	C	.	.	.	.	.	.	.	-	T	.	.	A	.	.	.	.	T	.	.	.	.	.	.	.	.	.	.
OU-SEBU	.	.	.	.	.	.	.	.	.	.	.	.	.	.	.	.	.	.	.	.	.	-	.	.	.	.	.	.	.	.	.	.	.	.	C	.	.	.	.	.	T
OU-DSRA	.	.	.	.	.	.	.	.	.	.	.	.	.	.	.	.	.	.	.	.	.	-	.	G	.	.	.	.	.	.	.	.	.	.	C	.	.	.	.	.	.
OU-DSME1	.	.	.	.	.	.	.	.	.	.	.	.	.	.	.	.	.	.	.	T	.	-	.	.	.	.	G	.	.	.	.	.	.	.	C	.	.	.	.	.	.
OU-SEUA	.	.	.	.	.	.	.	.	.	.	.	.	.	.	.	.	.	.	.	.	.	-	.	.	C	.	.	.	.	.	.	.	.	.	.	.	.	.	.	.	.
Hap. B	.	C	.	.	.	.	.	.	.	.	C	.	.	.	G	.	.	.	.	.	.	-	.	.	C	T	.	T	.	.	.	.	.	.	.	.	C	.	T	.	.
Hap. C	.	C	.	.	.	.	.	.	.	.	C	.	.	.	G	.	.	.	C	.	.	A	.	.	C	T	.	T	.	.	.	.	.	.	.	.	C	.	T	.	.
Hap. D	C	C	.	.	.	.	.	.	.	.	C	.	.	.	G	.	.	.	.	.	.	-	.	.	C	T	.	T	.	.	.	.	.	.	.	.	C	.	T	.	.
Hap. E	.	C	.	.	.	.	.	.	.	.	C	T	.	.	G	.	.	.	.	.	.	-	.	.	C	T	.	T	.	.	.	.	.	.	.	.	C	.	T	.	.
OU-TP6	.	C	G	.	.	.	.	.	.	.	C	.	.	.	G	.	.	.	.	.	.	-	.	.	C	T	.	T	.	.	.	.	.	.	.	.	C	.	T	.	.
OU-TP24	.	C	.	.	.	T	T	.	.	.	C	.	.	.	G	.	.	.	.	.	.	-	.	.	C	T	.	T	.	.	.	.	.	.	.	.	C	.	T	T	.
OU-TP15	.	C	.	.	T	.	.	T	.	.	C	.	.	.	G	.	.	.	.	.	.	-	.	.	C	T	.	T	.	.	.	.	.	.	.	.	C	.	T	.	.
OU-TNK41	.	.	.	.	.	.	.	.	.	.	.	.	G	.	A	.	.	.	.	.	.	-	.	.	C	.	.	T	T	.	.	.	T	.	.	.	.	.	.	.	.
OU-TNK36	.	.	.	.	.	.	.	.	.	.	.	.	G	.	A	.	.	.	.	.	.	-	.	.	C	.	.	T	T	.	.	.	.	.	.	.	.	.	.	.	.
OU-SO69	.	.	.	T	.	.	.	.	.	.	.	.	.	.	A	T	.	T	.	.	.	-	.	.	C	.	.	T	T	.	.	.	.	A	.	T	.	.	.	.	.
OU-KA1	.	.	.	.	.	.	.	.	.	.	.	.	.	.	A	.	.	.	.	.	.	-	.	.	C	.	.	T	T	.	.	.	.	.	.	.	.	.	.	.	.
OU-SB71	.	.	.	.	.	.	.	.	.	.	.	.	.	.	A	.	C	.	.	.	.	-	.	.	C	.	.	T	.	.	.	T	.	.	.	.	C	G	.	.	.
OU-SB70	.	.	.	.	.	.	.	.	.	G	.	.	.	.	A	.	C	.	.	.	T	-	.	.	C	.	.	T	.	.	.	T	.	.	.	.	C	.	T	.	.
OU-SB57	.	.	.	.	.	.	.	.	.	G	.	.	.	.	A	.	C	.	.	.	T	-	.	.	C	.	.	T	.	G	.	T	.	.	.	.	C	.	T	.	.
OU-SB60	.	.	.	.	.	.	.	.	.	.	.	.	.	.	A	.	C	.	.	.	.	-	.	.	C	.	.	T	.	.	.	T	.	.	.	.	C	.	.	.	.

References

1. Warren, K. S. *et al*. Speciation and intrasubspecific variation of Bornean orangutans, *Pongo pygmaeus pygmaeus*. *Mol. Biol. Evol.* **18,** 472–480 (2001).
2. Arora, N. *et al*. Effects of Pleistocene glaciations and rivers on the population structure of Bornean orangutans *(Pongo pygmaeus)*. *PNAS* **107,** 21376–21381 (2010).
